# Types of Economic Abuse in Postseparation Lives of Women Experiencing IPV: A Qualitative Study from Finland

**DOI:** 10.1177/10778012221127727

**Published:** 2022-09-29

**Authors:** Anniina Kaittila, Mia Hakovirta, Heini Kainulainen

**Affiliations:** 1 Department of Social Research, 8058 University of Turku, Turku, Finland; 2 Invest Flagship Research Center, University of Turku, Turku, Finland; 3 Office of the Non-Discrimination Ombudsman, Helsinki, Finland

**Keywords:** postseparation violence, economic abuse, IPV, postseparation economic abuse, divorce

## Abstract

There is growing interest in economic abuse as a form of violence against women, but the research has largely addressed cohabiting couples thus far, with few detailed explorations of women's experiences of economic abuse in postseparation life. Using interviews with 11 women experiencing intimate partner violence (IPV), this study investigates the types of economic abuse in the lives of these women. Inductive thematic analysis revealed four types of postseparation economic abuse (PSEA): economic sabotage, withholding resources, financial harassment, and stealing. These results help better understand and recognize the different forms of PSEA. Recommendations are provided for incorporating PSEA as a central component of IPV research, practice, and policy.

## Introduction

Intimate partner violence (IPV) is a social problem that crosses race, culture, society, socioeconomic status, and geographic environments. It has also been recognized as one of the most serious social and human rights issues (Humphreys, 2007; Postmus et al., 2020). IPV, understood as violence against women also views the manifestation of violence and its consequences as a structural issue, which undermines women's ability to function as full members of society. IPV takes many forms, such as physical, sexual, psychological, and economic abuse or violence. While the phenomenon of IPV has received ample consideration during recent decades, attention has been mainly focused on the first three forms of IPV listed above, with economic abuse remaining understudied

The economic abuse form of domestic violence has been explored more deeply in recent years (e.g., Natalier, 2018; Stylianou, 2018; Zeoli et al., 2013). International studies have reported the lifetime prevalence rates of economic abuse among women as 21% in the UK (Sharp-Jeffs, 2015) and 12% in Australia (Kutin et al., 2017), while there is little data available for the Nordic regions.

Economic abuse involves behaviors that control a woman's ability to acquire, use, and maintain economic resources; thus, the abuse threatens women's economic security and potential for self-sufficiency (Adams et al., 2020; Eriksson & Ulmestig, 2017; Postmus et al., 2020; Stylianou, 2018). Experiences of economic abuse have been associated with a range of negative outcomes among victims, including difficulty in gaining or maintaining employment (Swanberg & Logan, 2005), difficulty in establishing economic self-sufficiency (Voth Schrag, 2015), increased rates of depressive symptoms, and decreased rates of psychological well-being (Antai et al., 2014). In addition, economic abuse experiences impact family outcomes by decreasing stable family formations, parenting practices, children's behavior, and youth outcomes (Huang et al., 2015; Voth Schrag et al., 2016). Therefore, not only does the literature provide evidence for the impacts of economic abuse among IPV victims and their children, but it also documents the impact of economic abuse above and beyond other forms of IPV (Huang et al., 2015).

Previous research on economic abuse has largely addressed cohabiting or married couples (Adams et al., 2020; Postmus et al., 2020; Stylianou, 2018). Studies have shown that many IPV victims suffer economic abuse by their partners (Chowbey, 2017). Recently, it has been recognized that economic abuse may also occur after separation (e.g., Sanders, 2015; Ulmestig & Eriksson, 2017; Zeoli et al., 2013). It is reported that over 75% of abused women experience economic abuse (by their former spouses) in terms of withholding financial resources like child support, health insurance, and other basic expenses (Toews & Bermea, 2017). As women are at greater risk of stalking, violence, and intimate partner homicide when they attempt to leave abusive relationships (Ornstein & Rickne, 2013), men can use a variety of tactics to control their former spouses (Nikupeteri & Laitinen, 2015). Moreover, women are more likely to face economic hardships in postseparation life, which can lead to economic abuse (Kutin et al., 2017). Longitudinal research has also found that women who experienced IPV were substantially likely to experience material hardship during the 9-year follow-up period (O’Connor & Nepomnyaschy, 2020).

 Little is known about the forms and effects of postseparation economic abuse (PSEA), or women's experience of it. This study fills this research gap by examining, through qualitative interviews, the forms of economic abuse in the lives of women who have experienced IPV. The data consist of interviews of 11 Finnish women who were separated or divorced from IPV-perpetrating partners and experienced economic abuse. This study is guided by the research question: what types of PSEA can be found in the narratives of these women?

This study makes several contributions to the literature. First, economic abuse is significantly associated with other forms of IPV, but less is known about how financial and economic issues are visible in the postseparation lives of women. Second, most studies on PSEA are from the United States, Australia, and the United Kingdom (e.g., Chowbey, 2017; Tenkorang & Owusu, 2019; Ulmestig & Eriksson, 2017). Therefore, there is a need to study economic abuse in different welfare state contexts. Chowbey (2017) asserted that the characteristics of economic abuse are affected by social and cultural practices and are intersected with class, nationality, education, and employment status; thus, PSEA can emerge in unique forms in different contexts.

This study focuses on Finland, which is renowned for its gender equality, women-friendly welfare state, with universal social and health services and last-resort social safety nets (Kangas & Qvist, 2013). The welfare state context can influence women's exposure to economic abuse by increasing their abilities to establish independent households and allowing them to maintain strong ties to the labor market, which may ensure that they have the necessary resources to exit abusive relationships (Larsen, 2016). However, survey data suggest that the lifetime prevalence rates of IPV in Finland are among the highest in the European Union, which has been called the Nordic paradox of gender equality (Wemrell et al., 2019). Therefore, there is a need to explore the forms of PSEA in Finland.

## Literature Review

### Economic Abuse as a Form of IPV

According to Stylianou et al. (2013), economic abuse can be divided into three categories: economic exploitation, economic control, and employment sabotage. Economic exploitation occurs in situations where the abuser either depletes a woman’s funds or commits acts that result in debt or ruin their credit (Adams et al., 2008; Seith, 2001). Economic control refers to the abuser monitoring and restricting a woman's use of her own or her family's common resources; that is, the abuser isolates a woman from her financial resources. For example, a man may take control of all decision-making that concerns finances or may demand that a woman transfers all her income for the man to monitor and use (Sanders, 2015; Usta et al., 2013).

In employment sabotage, the abuser restricts a woman's ability to obtain her own resources through employment. For example, a man may harass a woman's workplace by calling or entering her workplace repeatedly or by stalking her during the workday (Galvez et al., 2011; Pyles & Banerjee, 2010). Another form of employment sabotage is limiting a woman's employment, where a man may take car keys or bus passes so that it is impossible for a woman to get to her workplace, or he may physically abuse her so that it is physically impossible for her to work (Brush, 2011; Ficco, 2007).

Economic and financial issues play a role in IPV. Stylianou et al. (2013) studied an overlap in reporting physical, emotional, and economic abuse of violence experiences, and found that 75.8% of participants reported experiencing all forms of IPV. Violence overlap has also been observed in other studies (Anderson, 2010; Basile & Hall, 2011). In addition, these violent actions may take place simultaneously; for example, a man may steal a woman's money by threatening her with physical violence. Sanders (2015) reported that most women interviewed believed that financial factors played a significant role in their experiences of IPV. Further, economic abuse can also be a risk factor in women's lives by increasing their vulnerability to physical violence, sexual abuse, and other criminal activities (Fawole, 2008). Sanders (2015) further argued that there are several intersections between economic factors and IPV intermediates. Women with low incomes are vulnerable to abuse and are often prevented from leaving abusive partners due to economic dependence (Wilcox, 2006). Thus, women's economic status and their ability to obtain or maintain employment are significantly associated with IPV.

### Postseparation Economic Abuse

Previous studies on economic issues and abuse have mostly focused on partnered women (e.g., Chowbey, 2017; Huang et al., 2015; Sanders, 2015; Krigel & Benjamin, 2020). However, financial and economic matters are also visible in postseparation life (Natalier, 2018; Toews & Bermea, 2017; Ulmestig & Eriksson, 2017) and can occur in the same form as economic abuse during a relationship; however, in the postseparation context, economic abuse is specifically understood as the continuous act of causing financial harm to a partner, even after separation has occurred (Natalier, 2018). In postseparation life, many women are economically vulnerable, which builds and reproduces hierarchical relations of power that exuberate risk for economic control and abuse. Therefore, from a feminist perspective, IPV may be explained by a man's desire to control a woman, even when separated (e.g., Cook & Natalier, 2013; Natalier, 2018). Economic abuse can also become more critical after separation and divorce, when abusive partners search for revenge or punishment (Krigel & Benjamin, 2020).

Men can use various tactics to control their former spouse in postseparation life. The most used tactics are harassment and intimidation, undermining a mother's ability to parent, discrediting a former wife as a mother, withholding financial support, endangering children, disregarding children, disrupting a mother's relationship with her children, and using physical/sexual violence against a former wife and her children (Hardesty et al., 2008; Humphreys & Thiara, 2003; Toews & Bermea, 2017; Zeoli et al., 2013). The most documented form of PSEA is withholding financial resources (Toews & Bermea, 2017).

 In postseparation life, withholding behavior is an example of economic control because it enables former partners to restrict women's access to and monitor their use of economic resources (Natalier, 2018). In Toews and Bermea’s (2017) study, over 75% of the interviewed abused women reported that their former husbands withheld financial resources such as child support, health insurance, and other basic expenses that they had already agreed to pay. They concluded that the violent and coercive behaviors men used during marriage continued to influence the women's perceptions of the power and control that their former husbands had over them in postseparation life.

Ulmestig and Eriksson (2017) studied how survivors of domestic violence experienced financial vulnerability and the implications of these experiences on social work within the social assistance system in Sweden. Based on 13 in-depth interviews with female survivors, they showed how debts and stolen money, together with difficulties in the labor market, affected women's ability to have a reasonable economic standard in postseparation life.

Threatening ex-spouses’ employment and taking money without permission from a woman's bank account are typical forms of postseparation abuse. Jaffe et al. (2003) showed that women who had experienced IPV reported that their former husbands had ruined them financially, hidden money from them, taken money from their bank accounts, or quit jobs to reduce their child support payments. It has also been found that men sometimes destroyed their ex-spouses’ possessions to weaken their financial situation (Brewster, 2003).

Previous research has documented a range of economically abusive behaviors that perpetrators use to threaten their partners’ economic stability. As these partners do not live together, PSEA can also have its own characteristics and forms. Douglas (2018) found that legal processes may provide an opportunity for perpetrators to both continue and expand their repertoire of control and abuse. Hiding money and manipulating assets are typical forms of postseparation abuse. For example, men may hide assets before property settlement so there would be less money available for their ex-spouse.

Withholding and delaying child support have been recognized as a PSEA strategy. Delaying child support payments can be a means for men to weaken the financial situation of their ex-spouse (Jaffe et al., 2003). Sometimes, this abusive behavior continues for years, where men pay less child support than their income would suggest them capable of. The research has also highlighted the ways in which men underreport their income, withhold child support, or request multiple changes of assessment as part of a broader campaign of control over their former partners (Natalier, 2018). This has been particularly documented in men who are self-employed or who work in family businesses (Toews & Bermea, 2017). Inaccurate recording of income and contact patterns also provides an inaccurate basis for the calculation of individual women's child support assessments and often results in the determination of lower child support liabilities than would otherwise be required (Cook & Natalier, 2013). The deliberate withholding of child support is a form of economic abuse that is facilitated through gendered state processes and institutions that order child support transfers. Masculine financial discretion structures policy and organizational practices legitimize men's financial agency at the expense of women's financial autonomy (Natalier, 2018). Thus, gaps in policy and law can exacerbate the problem by offering insufficient protection against an ex-spouse who refuses to deliver the agreed child support or deliberately prolonging custody disputes to harm a woman economically (see also Elizabeth et al., 2012; Watson & Ancis, 2013).

## Methodology

### Sample Description

 For the analysis, we combined two research projects to develop the types of economic abuse in the postseparation lives of women experiencing IPV. Study one (S1) was an international research project titled: “Mapping the legislation and assessing the impact of Protection Orders in the European Member States” (Van der Aa et al., 2015). Structured interviews were conducted with women who had experienced IPV and sought protection orders. The selection criteria for participants were: (1) female victims who had (2) experience of domestic violence and/or stalking by an ex-spouse, (3) had obtained a criminal protection order against their male ex-spouse, (4) as a result of a specific, quasi-criminal protection order procedure, (5) in reaction to an incident that happened less than four years ago. The interviews took place from 2013 to 2014. The participants were recruited from the Federation of Mother and Child Homes and Shelters (child welfare organization) and Women's Line (help-line for violence), which had published an announcement of the study on their bulletin boards, webpages, and email lists. In addition, a letter was sent to 52 women who had gained a protection order by the District Court of Helsinki or Espoo (the capital area) from 2011 to 2013. The announcement and the letter briefly explained the study and its purpose and asked participants to contact the researcher. The interviewers did not ask how the participants found out about the study (Van der Aa et al., 2015). A total of 16 women participated in this study.

 Study two (S2) was a research project concerning the experiences of help-seeking, juridical processes, and economic abuse. It was a domestic project in Finland. The participants were recruited from Women's Line, the Federation of Mother and Child Homes and Shelter, and one person was recruited from a personal contact. In S2, all participants were rewarded with a ticket to a movie theater in recognition of their time and effort to participate. Interviews were conducted between 2014 and 2015. A total of 7 women participated in this study.

In both studies, most interviews were conducted at the University of Turku or the University of Helsinki, while some were conducted via the Internet. The data was collected by three interviewers (one in S1, two in S2). Two of the interviewers had experience with research on IPV, but not specifically in economic abuse. The third of the interviewers had explored economic abuse in her previous studies. Interviews that contained narratives of PSEA were included in the data. Informed consent forms were obtained from all respondents. The final data consisted of 11 interviews with women who had experienced PSEA. Nine of these interviews were from S1 and two interviews were from S2. The women were aged between 31 and 46 years. Nine of the women had children, and the number of children varied from two to four. In six of the cases, the abusive ex-spouse was also the father of the children. Six of the women were married to men who were violent toward them, five were cohabiting, and one was dating and living in a separate apartment. Ten women had Finnish as their native language and one had Swedish. The women's level of education varied from vocational schools to master’s degrees.

### Interview Protocol

In S1, interviews were conducted using a semistructured interview protocol. The questions were mostly open-ended and covered all aspects of the protection order procedure through which problems could arise for victims. The semistructured design was chosen to allow victims to elaborate on certain issues or to bring up new issues if needed. If something important came up, the interviewers could remain focused on the issue and investigate the matter further. There was a basic questionnaire protocol containing questions on the history of the violence that the victim had experienced, the incident that eventually led to the protection order, the procedure through which the protection order was imposed, the way that the police and the public prosecution service had treated the victim, the effect of the protection order on the violence experienced, and the victim's satisfaction with the protection order (procedure).

 In S2, narrative interviews were used, and the focus was on the victims’ experiences and temporality. The interview contained three key themes: experience of the criminal procedure, experience of help-seeking and receiving, and experience of economic abuse. These themes contained a loose key question (i.e., “Could you describe the situation in which you first contacted the police” in the first theme) at the beginning, and the aim was to allow for participants’ own speech. This narrative method was clarified with follow-up questions, and probes were used to elicit details about the victims’ individual experiences. All interviews in S1 and S2 were conducted in Finnish, audio recorded, and then transcribed.

### Data Analysis

To manage and analyze the data generated through the interviews, the qualitative software package NVivo was utilized. The analysis was informed by Braun and Clarke's (2006) inductive thematic analysis, and preliminary themes were defined from the data without utilizing any preexisting coding framework. First, the interviews were read carefully, and content that contained descriptions of postseparation situations and financial matters was selected as the final data by A.K. To reinforce the reliability of the data selection, researchers A.K. and M.H. read the interviews and checked that all the material that described PSEA were involved in data. Second, A.K. line-coded descriptions using an open-coding technique. The codes were discussed, specified, and accepted by the research group (A.K., M.H., and H.K.). Third, A.K. distributed these codes into subthemes, which she then grouped into larger categories. In a meeting with the authors (A.K., M.H., and H.K.), the analysis process was elaborated and subthemes and larger categories were discussed. The researchers agreed that the analysis was conducted carefully and categories represented the data.

Throughout the studies, the authors followed the principles of staying alert and ethically sensitive throughout and sought to reflect on their actions throughout the writing process (Notko et al., 2013). The research plan (S2) was approved by the Ethical Board of the University of Turku. The data were anonymized by using pseudonyms and other information that would not enable the identification of the interviewed women.

### Economic Abuse in Women's Postseparation Life

Based on the interviews, PSEA could be divided into four categories: (1) economic sabotage, (2) withholding resources, (3) financial harassment, and (4) stealing. Economic sabotage encompasses behavior that destroys the possessions of the victim or prevents them from obtaining or maintaining employment. Withholding resources refers to behavior where the abuser limits the victim's access to financial resources, such as property or child support. Financial harassment involves harrying victims over financial issues, while stealing refers to taking the victim's property without permission or approval. While the boundaries between the categories are clearly defined, they are also connected, and, to some extent, overlap. The contents of these forms of PSEA are presented in Table 1.

**Table 1.** Forms of PSEA Among Women Experiencing IPV*.*

Economic sabotage	Withholding resources	Financial harassment	Stealing
Destroying possessions.	Prolonging the divorce process and refusing to divide assets.	Futile and false accusations.	Refusing to return a woman's goods.
Sabotaging employment.	Refusing to pay one's share of bills or purchases.	Using financial issues to keep in contact.	Stealing a woman's property.
	Denying access to women's property.	Threatening social networks.	
	Withholding child support payments.		

### Theme 1: Economic Sabotage

The first theme to emerge when discussing the role of financial matters in the women's experiences of IPV was economic sabotage, which refers to the destruction of their possessions. Several women revealed that they had experienced economic sabotage at the hands of their ex-spouses. On some occasions, this meant the destruction of their property, such as their house or personal belongings. Though the women had not always witnessed their ex-spouses’ sabotage, they were convinced that the acts were conducted by them because of the context. The acts happened after other forms of contact from the ex-spouses.

 The women revealed two types of destruction: regarded and impulsive. Regarded destruction was often ongoing and happened without the women seeing the act. This type of destruction also involved monitoring the women. Elena describes her experience as follows:

The car was violated and smashed. A number of things could not be proved, but I was quite certain who was behind it. This happened quite oftenmonthly.

Impulsive destruction occurred as revenge for something the women had said or done. Some women were at home during the violent act, while others were not. If a woman was at home, the destruction of her belongings happened together with an attempt to violate her physically. In such a situation, it was common for a woman's property to be broken when, for example, a man threw goods or otherwise broke objects. For Sanna, her ex-spouse's main aim was not to destroy her belongings, yet this happened because he was trying to prevent her from calling the police, as follows:

I was calling the police when he caught me and threw my phone at the wall of the house next door. I escaped and ran inside. He threatened to kill me. I hid in the children's bathroom and called the emergency center using the home phone. He tried to break down the door, so I jumped out of the toilet's small window on the second floor. The police arrived and found him half naked looking after me … He had broken the bathroom door.

In some cases, the men wanted to make sure that in the situations in which the women stayed at the house or apartment that the ex-couple had lived in during their relationship, the women would not have the possibility to use or enjoy the belongings that the man had bought. These situations took extreme forms for Riitta, when her ex-spouse was last in the house that Riitta owned but had lived in together. Her ex-spouse wanted to rip off the sauna stove that he had bought. This example represents the cultural meaning of belonging, since sauna bathing in Finland is a cultural heritage with a long history. She stated that:

When I was taking a shower, he ripped off the sauna stove and caused burns to his body. He wanted to take the sauna stove with him so that I was not able to enjoy it. When the police came, he was very angry. The police said that he could not take any structural elements of the house with him. However, he wanted it because he said that he had bought it.

In some cases, the ex-spouse harassed the women's employment or destroyed their workplaces. For Rosa, this harassment was extensive, with constant phone calls to her workplace and pictures and notes posted to the workplace door. It became impossible for her to run her business, and she had to terminate it. She stated that:

Odd things started to occur. I had my own company, and there soon started to appear all kinds of photos and prostitution messages, such as “Services for men in the back room,” posed on the door of my business. He also posted a photo of us on my business door. He called my workplace every three minutes and sent some horrific text messages. There was a large number of messages. At first I answered him, and he made financial demands. Finally, I ended my business, moved to another city, and received a protection order; then the harassment ended.

### Theme 2: Withholding Resources

The second theme to emerge was the men's urge to control the women via withholding resources. While within the theme of economic sabotage men were actively doing something with withholding men withheld or refused to do things. For example, they delayed divorce, refused to divide assets, refused to pay their share of bills or purchases, or refused to move out of the woman's apartment.

Elina's case shows how ex-spouses may delay the finalization of divorce in Finland by ignoring requests to sign divorce documents. In Finland, either or both of the spouses may file for divorce, and the application is filed at a district court. If spouses are filing for divorce, the reconsideration period begins as soon as the application is filed. If one of the spouses is filing for divorce alone, then the reconsideration period begins once the other spouse has been notified of the divorce application. The district court will ensure that the other spouse is notified of the application (Marriage Act, 1929). This policy may delay the process, as shown in the following:

He did not want to sign divorce papers. I am still married to him because he will not give me a divorce. It seems that in Finland, it takes two years before it is possible to get rid of someone. The divorce would otherwise be clear, and the papers have been signable since May. A bailiff has tried to reach him since July. In September, I contacted him via text message and asked if he could sign the divorce papers. He texted back that I should come in person to ask for a signature for the divorce papers.

When Helena decided to separate from her spouse, he refused to accept the divorce and used financial resources to state that the relationship had not ended. In her case, refusing to divide their property in the divorce had both economic and psychological consequences. Being legally tied to an ex-spouse made it more difficult to psychologically move forward with her life and to complete the division of property that the law requires after divorce. She stated:

Our divorce became legal, but my husband disagreed to divide the property. We do not have any assets to share. I just would have wanted to sign it on paper with a date. He never agreed to do that because he said that the relationship was not over. He did not believe it was. At some point, I did it by myself and wrote a letter that the property has been divided into two apartments. There was a letter with our names and the date, but he refused to sign it; therefore, the division of property is still incomplete.

Economic abuse can also deny a victim access to their property. In Laura's case, her ex-spouse decided that he would not hand over her possessions. This case shows how financial matters during and after relationships are tied together, and that unequal practices during the relationship are also present when separating. According to Laura, her ex-spouse systematically accumulated his own property during their relationship. They shared a common corporation with her ex-partner. The company made a profit, but her ex-spouse took all the money available from the corporation and used them for his own expenses. In addition, they bought an apartment together with the agreement that Laura owned 20% and he owned 80%. Nevertheless, during the relationship, both paid the same amount for the loan and the apartment's common expenses. Laura separated from her ex-spouse a year earlier and has since argued with her ex-partner. According to Laura, her ex-partner did not want Laura to live in an apartment they used to share. In addition, he refused to acknowledge and compensate her for the apartment. She stated:

He has increased his property at my expense. This is a big issue right now. We are trying to sort it out with lawyers, police, and the executors of the property. Now, we just have to go to court.

In PSEA, children can be used as an excuse to withhold resources from women. Child custody, child contact, and child support arrangements enable women to be harmed financially. It is noteworthy that these situations harm not only women, but also children. Men may refuse to pay for children's expenses or child support. In Erika's case, she had child support agreements ratified by the social welfare board, yet her ex-spouse refused to pay child support, which left a substantial gap in Erika's income. It did not affect only her, but also their children's financial well-being. Erika had to sue her ex-spouse and pursue child support through the court. This case shows how men can use family law and the child support process as a way of directly or indirectly controlling their former spouse and undermining their financial security and self-reliance.

Starting a new life after leaving a violent relationship often requires finding a new home. This may not be easy, since there are few reasonably priced rental apartments, private dormitory housing is expensive, and the women's financial situation in postseparation life is often challenging. The situation becomes even more difficult, as in Leena's case, when her ex-spouse controlled her by refusing to move out of their apartment. Leena also suggested that she could move away and acquire a new apartment, but her ex-spouse did not agree to pay the rent for the apartment he would be living in. It was not possible for Leena to move into a new apartment and pay two different rents simultaneously. Therefore, she was forced to live with her ex-spouse, who had been violent toward her, for several months until the rental agreement ended.

### Theme 3: Financial Harassment

Through sabotage, men harm women's property and employment, by withholding men withheld or refusing to do something that was vital for the women. With harassment, men use financial matters to bully and control women and make accusations concerning finances. Men may indicate that the woman has stolen his belongings or may bully her with financial issues using other means. From the women's interviews, it is evident that when a couple has lived together, it becomes very difficult to prove whose goods belong to whom. Veera described her experience when her ex-spouse came to her house, where they had lived as a couple, as follows:

I collected all his belongings—a big lawnmower, bikes, hobby equipment, and clothes—and left them all in the same place. I then left the house because I did not want to meet him in person. He took all the things to his van and started to text me. You missed this and that personal possession that did not even exist, and things that he really did not own.

The women's narratives emphasize how money and financial issues work as excuses to maintain contact in postseparation life. In these examples, money may not be used as the main reason for the men to make contact, but rather as an instrument to make a point. Heta describes how her ex-spouse did not want to separate and he wowed love, but at the same time threaten how Heta would not make it financially if she will end the relationship. In situations where women's financial situations are difficult, such messages may challenge the decision to leave the abusive relationship, as follows:

He kept texting me saying how he missed and loved me. He also threatened that if I pursue a divorce, I will experience financial distress, which I already knew. We shared finances as a couple.

In Rosa's situation, the harassment was not limited to her but also her personal network and her new boyfriend. Rosa's ex-spouse asked her new boyfriend to pay “leasing” due to being with her. In her experience, the characteristics of money became visible, as money was not only a means of payment but was also a tool to control and place oneself over the other.

The women's narratives show how economic abuse continues over time. Katja's example demonstrates how economic abuse is often, though not always, started during the relationship and continues in postseparation life. In addition, financial agreements made during the relationship also regulate postseparation life. For Katja, these agreements have caused her enormous financial harm, but common financial matters have also given her ex-spouse an excuse to contact her years after their separation, as follows:

He is self-employed, but everything is in my name: all ownership, including companies, and other things. He has taken advantage of me in financial matters and uses it against me (in this process). He talked me out of it all, and I still suffer from the fact that I must sign all the papers. He used my money, and, even after the separation, there are messages saying pay for this and do this.

### Theme 4: Stealing

Stealing is a form of economic exploitation when an abuser intentionally engages in behaviors aimed at destroying the victim's financial resources. The interviews revealed situations in which the men stole from the women. Compared to the other categories, in stealing, the men actively took something that belonged to the women. For example, Laura left her ex-spouse and moved out of his house. The ex-spouse protested her decision, changed the locks of the house, and stole her movies, which had great sentimental value for her, as follows:

There were hundreds of my movies and all kinds of materials. I collected them for over 10 years, and I'd love to get them back. They are worth money, and the emotional value is incredible. I do not want to give them to him as he has taken everything else from me.

For Laura, it was important to gain back her belongings, and she had filed a criminal complaint against her ex-spouse because of the theft. In some situations, as in Sirpa's case, the women felt that defining one's rights using goods would be life-threatening and may cause them substantial financial harm. In Sirpa's situation, she did not have enough emotional resources or energy to exert herself, even though she was left financially disadvantaged and lost mentally important goods. From Sirpa's case, it was clear that keeping herself safe from her ex-spouse's controlling behavior and violence was more important than defending her property, as follows:

My ex-husband keeps his motorbike in my courtyard. When we divorced, he took my motorbike with him, and I could not oppose it forcefully. I would rather be alive and without a motorbike than with a motorbike and dead.

## Discussion

This study presents qualitative findings of the types of economic abuse in the postseparation lives of women who have experienced IPV. From the women's narratives, their experience of PSEA was divided into four categories: economic sabotage, withholding resources, financial harassment, and stealing. The ex-spouses sabotaged the women financially and destroyed their belongings both inside and outside of their homes or workplaces. It was evident that abuse occurred in important places for the women; however, as Wilcox (2006) stated, one's home is supposed to be relatively permanent and safe, and women are assumed to have control there. Therefore, this assumption provides a sense of security and stability that domestic violence disrupts.

In previous studies, one of the most documented forms of postseparation abuse is by withholding resources (Natalier, 2018; Toews & Bermea, 2017). This type of violence was also highlighted in the current study. Withholding resources meant refusing to divide assets, withholding women's possessions postseparation, refusing to pay one's share of bills or purchases, delaying divorce, and refusing to leave apartments. Importantly, divorce and family law practices have enabled PSEA against women (see also Natalier, 2018); for example, the system allows men to prolong the divorce process and “play” with child support. As child support often evolves in the private sphere (Skinner et al., 2007), controlling practices are well hidden, and the involvement of support services may not be obvious, while the absence of formal legislation may exacerbate this. These situations can cause substantial financial harm for women and their children, as child support compliance is a problem in many countries (Hakovirta & Mesiäislehto, 2022).

 The women also experienced financial harassment. This meant bullying over money-related issues, such as claiming women's property and reminding women how they would fall into financial crisis if they ended a relationship. Some women had also experienced theft; in these cases, the women lost mentally and financially valuable belongings. Indeed, the types of PSEA discovered herein demonstrate that economic abuse both during a relationship and in postseparation life have common and differing characteristics. Adams et al.'s (2008) pioneering study concluded that financial abuse occurs when the offender interferes with the victim's ability to acquire, use, or maintain financial resources in different ways. All these elements are also transparent with PSEA; however, the acts of economic abuse may also mean bullying and harassment over financial issues.

 The women's narratives presented, as is typical with PSEA, not certain behaviors per se, but rather a sum of acts that may vary by their nature. Indeed, this study supports the evidence from the previous studies that suggest that postseparation violence is often continuous, and takes several forms (Toews & Bermea, 2017). Moreover, the current study's results suggest that PSEA can be unique in form but is also linked to physical, emotional, and digital violence and/or stalking of women. The interviewed women often experienced several forms of violence, and the acts of economic abuse often contained other forms of violence or followed violent acts, such as destroying the women's possessions after stalking them.

A broad agreement exists about the negative economic consequences of union dissolution for women (e.g., De Vaus et al., 2017). This may expose PSEA, as women who have low economic resources are at a greater risk of abuse (Wilcox, 2006). Thus, postseparation economic inequality reproduces the hierarchical relations of power that exuberate risk for economic control and abuse. Sometimes, PSEA can lead to financial insecurity for economically vulnerable women, as women must spend money to repair their broken possessions or cover their lawyers’ expenses. In addition to financial insecurity, PSEA causes substantial harm to women's mental well-being.

The findings of this study represent the context of Finland, which is renowned for its women-friendly welfare state as a part of the Nordic welfare model, but where the prevalence of violence against women is relatively high (Wemrell et al., 2019). The current study demonstrates the importance of situating PSEA in a sociolegal context, which on the one hand affects how abusers may use institutional processes and practices to harm women, and on the other hand protects the victims of PSEA, for instance providing social services and enabling help-seeking. However, IPV is a fairly universal phenomenon (see Devries et al., 2013). It can take very similar forms across nations and therefore the types of PSEA presented in this study may also occur in different welfare state contexts.

### Implications

This study's results help to understand and recognize various forms of PSEA. This information is vital for educating social workers and healthcare professionals to observe and intervene in economic abuse, and to develop services for the victims. This study argues that PSEA should be considered within the different spheres of social policy, legislation, and social work practices. From the results of this study, it is clear that social policies and legal practices, to some extent, presume that spouses negotiate and agree upon practices, such as divorce and child support, in a mutual understanding and without elements of violence, harassment, or control (see also Branigan, 2004). Therefore, it is vital to properly analyze how these practices acknowledge the possibility of violence, and necessary improvements should be made.

 At the clinical level, there are well-structured measurements and methods to assess the types and extent of economic abuse within relationships (e.g., Adams et al., 2020; Surviving Economic Abuse, 2021). However, to the best of this study's knowledge, there is insufficient material to evaluate the presence of PSEA. This study suggests that such measurements should be developed for this phenomenon and that it would be useful to implement them in different services for individuals and separated families, such as social work, child welfare supervision, counseling, and therapy. In cases where economic abuse is acknowledged, there is often a substantial need for multiprofessional collaboration, since, according to the data herein, there is a simultaneous need for legal support, psychological support, and financial counseling (see also Notko et al., 2021). Thus, multiagency collaboration should be developed.

### Limitations and Future Directions

The results of this study should be interpreted in light of certain limitations. It should also be noted that in the data of this study, the focus was not explicitly on PSEA, and therefore it may have missed some important elements of PSEA accordingly. Indeed, all the women had experienced IPV during their relationships and there were none who had only experienced violence in postseparation life, although it is known that there are relationships where violence starts in postseparation life. The recruitment of participants via shelters, helplines, and directly to women who had received protection orders influenced the composition of the current study's sample in a way that the respondents were active in seeking help and support. Therefore, it is likely that the current study's sample lacks inclusion of the most vulnerable women who have basic language skills and a limited understanding of the welfare system (e.g., Ulmestig & Eriksson, 2017). Thus, the results reflect Finland's sociocultural and sociolegal contexts, yet in different contexts, there would be somewhat different experiences with PSEA. In addition, we need to acknowledge that working across two small-scale interview data interview questions addressed to interviewees were not similar between the projects. However, despite the differences between the two studies, they offered a very similar picture of PSEA and its consequences.

Since PSEA has gained only limited attention, it is vital to explore its characteristics, prevalence, and consequences. In addition, further research should clarify how institutional contexts (i.e., service, legal, and policy) either respond to the needs of victims of PSEA or enable violence. Since the characteristics of economic abuse are affected by the sociocultural context, country-level comparisons in future studies will be important. As we move forward, research on economic abuse in postseparation life needs to increase to develop practices and policies that are aimed specifically at PSEA. This suggests the need for ongoing research in this area.

## References

[bibr1-10778012221127727] Adams A. E. Greeson M. R. Littwin A. K. Javorka M. (2020). The revised scale of economic abuse (SEA2): Development and initial psychometric testing of an updated measure of economic abuse in intimate relationships. Psychology of Violence, 10(3), 268–278. 10.1037/vio0000244

[bibr2-10778012221127727] Adams A. E. Sullivan C. M. Bybee D. Greeson M. R. (2008). Development of the scale of economic abuse. Violence Against Women, 14(5), 563–588. 10.1177/1077801208315529 18408173

[bibr4-10778012221127727] Anderson K. L. (2010). Conflict, power, and violence in families. Journal of Marriage and Family, 72(3), 726–742. 10.1111/j.1741-3737.2010.00727.x

[bibr5-10778012221127727] Antai D. Oke A. Braithwaite P. Lopez G. B. (2014). The effect of economic, physical, and psychological abuse on mental health: A population-based study of women in the Philippines. International Journal of Family Medicine, 2014, 852317. 10.1155/2014/852317 25525517 PMC4265545

[bibr6-10778012221127727] Basile K. C. Hall J. E. (2011). Intimate partner violence perpetration by court-ordered men: Distinctions and intersections among physical violence, sexual violence, psychological abuse, and stalking. Journal of Interpersonal Violence, 26(2), 230–253. 10.1177/0886260510362896 20410373

[bibr7-10778012221127727] Branigan E. (2004). His money or our money? Financial abuse of women in intimate partner relationships. A report for the Coburg-Brunswick Community Legal and Financial Counselling Centre. http://csmc.org.au/wp-content/uploads/2014/05/Financial_Abuse.pdf

[bibr8-10778012221127727] Braun V. Clarke V. (2006). Using thematic analysis in psychology. Qualitative Research in Psychology, 3(2), 77–101. 10.1191/1478088706qp063oa

[bibr9-10778012221127727] Brewster M. P. (2003). Power and control dynamics in pre-stalking and stalking situations. Journal of Family Violence, 18(4), 207217. 10.1023/A:1024064214054

[bibr10-10778012221127727] Brush L. D. (2011). Poverty, battered women, and work in U.S. public policy. Oxford University Press.

[bibr11-10778012221127727] Chowbey P. (2017). Women's narratives of economic abuse and financial strategies in Britain and South Asia. Psychology of Violence, 7(3), 459–468. 10.1037/vio0000110

[bibr12-10778012221127727] Cook K. Natalier K. (2013). The gendered framing of Australia’s child support reforms. International Journal of Law, Policy, and the Family, 27(1), 28–50. 10.1093/lawfam/ebs013

[bibr13-10778012221127727] De Vaus D. Gray M. Qu L. Stanton D. (2017). The economic consequences of divorce in six OECD countries. Australian Journal of Social Issues, 52(2), 180–199. 10.1002/ajs4.13

[bibr14-10778012221127727] Devries K. M. Mak J. Y. García-Moreno C. Petzold M. Child J. C. Falder G. Lim S. Bacchus L. J. Engell R. E. Rosenfeld L. Pallitto C. Vos T. Abrahams N. Watts C. H. (2013). Global health. The global prevalence of intimate partner violence against women. Science (New York, N.Y.), 340(6140), 1527–1528. 10.1126/science.1240937 23788730

[bibr15-10778012221127727] Douglas H. (2018). Legal systems abuse and coercive control. Criminology & Criminal Justice, 18(1), 84–99. 10.1177/1748895817728380

[bibr16-10778012221127727] Elizabeth V. Gavey N. Tolmie J. (2012). ''…He's just swapped his fists for the system'': The governance of gender through custody law. Gender & Society, 26(2), 239–260. 10.1177/0891243211434765

[bibr17-10778012221127727] Eriksson M. Ulmestig R. (2017). “It’s not all about money”: Toward a more comprehensive understanding of financial abuse in the context of VAW. Journal of Interpersonal Violence, 36(3–4), 1625–1651. 10.1177/0886260517743547 29295038

[bibr18-10778012221127727] Fawole O. I. (2008). Economic violence to women and girls: Is it receiving the necessary attention? Trauma, Violence, & Abuse, 9(3), 167–177. 10.1177/1524838008319255 18495936

[bibr19-10778012221127727] Ficco D. M. (2007). Women stepping out: Intersections of welfare policy, work, and abuse [Unpublished doctoral dissertation]. The University of Pittsburgh.

[bibr20-10778012221127727] Galvez G. Mankowski E. S. McGlade M. S. Ruiz M. E. Glass N. (2011). Work-related intimate partner violence among employed immigrants from Mexico. Psychology of Men & Masculinity, 12(3), 230–246. 10.1037/a0022690

[bibr21-10778012221127727] Hakovirta M. Mesiäislehto M. (2022). Lone mothers and child support receipt in 21 European countries. Journal of International and Comparative Social Policy, 38(1), 36–56. 10.1017/ics.2021.15

[bibr22-10778012221127727] Hardesty J. L. Khaw L. Chung G. H. Martin J. M. (2008). Coparenting relationships after divorce: Variations by type of marital violence and fathers’ role differentiation. Family Relations: An Interdisciplinary Journal of Applied Family Studies, 57(4), 479–491. 10.1111/j.1741-3729.2008.00516.x

[bibr23-10778012221127727] Huang C. C. Vikse J. H. Lu S. Yi S. (2015). Children’s exposure to intimate partner violence and early delinquency. Journal of Family Violence, 30(8), 953–965. 10.1007/s10896-015-9727-5

[bibr24-10778012221127727] Humphreys C. (2007). A health inequalities perspective on violence against women. Health and Social Care in the Community, 15, 120–127. 10.1111/j.1365-2524.2006.00685.x 17286673

[bibr25-10778012221127727] Humphreys C. Thiara R. K. (2003). Neither justice nor protection: Women's experiences of post-separation violence. Journal of Social Welfare and Family Law, 25(3), 195–214. 10.1080/0964906032000145948

[bibr26-10778012221127727] Jaffe P. G. Crooks C. V. Poisson S. E. (2003). Common misconceptions in addressing domestic violence in child custody disputes. Juvenile & Family Court Journal, 54(4), 57–68. 10.1111/j.1755-6988.2003.tb00086.x

[bibr27-10778012221127727] Kangas O. Qvist J. (2013). Nordic welfare states. In Greve B. (Ed.), The Routledge handbook of the welfare state (pp. 124–136). Routledge Handbooks Online. 10.4324/9781315207049-13.

[bibr28-10778012221127727] Krigel K. Benjamin O. (2020). From physical violence to intensified economic abuse: Transitions between the types of IPV over survivors’ life courses. Violence Against Women, 27(9), 1121–1231. 10.1177/1077801220940397 32672102

[bibr29-10778012221127727] Kutin J. Russell R. Reid M. (2017). Economic abuse between intimate partners in Australia: Prevalence, health status, disability, and financial stress. Australian and New Zealand Journal of Public Health, 41(3), 269–274. 10.1111/1753-6405.12651 28245514

[bibr30-10778012221127727] Larsen M. (2016). IPV From a welfare state perspective. In Health inequities related to intimate partner violence against women (pp. 31–58). Springer. 10.1007/978-3-319-29565-7_3

[bibr31-10778012221127727] Marriage Act, § 234 (1929). https://www.finlex.fi/fi/laki/kaannokset/1929/en19290234_20011226.pdf

[bibr32-10778012221127727] Natalier K. (2018). State facilitated economic abuse: A structural analysis of men deliberately withholding child support. Feminist Legal Studies, 26(2), 121–140. 10.1007/s10691-018-9376-1

[bibr33-10778012221127727] Nikupeteri A. Laitinen M. (2015). Children's everyday lives shadowed by stalking: Post-separation stalking narratives of Finnish children and women. Violence & Victims, 30(5), 830–845. 10.1891/0886-6708.VV-D-14-00048 26299800

[bibr34-10778012221127727] Notko M. Husso M. Piippo S. Fagerlund M. Houtsonen J. (2021). Intervening in domestic violence: Interprofessional collaboration among social and healthcare professionals and the police. Journal of Interprofessional Care, 36(1) 15–23. 10.1080/13561820.2021.1876645 33657958

[bibr35-10778012221127727] Notko M. Jokinen K. Kuronen M. Pirskanen H. Malinen K. Harju-Veijola M. (2013). Encountering ethics in studying challenging family relations. Families, Relationships and Societies, 2(3), 395–408. 10.1332/204674313X665085

[bibr36-10778012221127727] O’Connor J. Nepomnyaschy L. (2020). Intimate partner violence and material hardship among urban mothers. Violence Against Women, 26(9), 935–954. 10.1177/1077801219854539 31304881

[bibr37-10778012221127727] Ornstein P. Rickne J. (2013). When does intimate partner violence continue after separation? Violence Against Women, 19(5), 617–633. 10.1177/1077801213490560 23743350

[bibr38-10778012221127727] Postmus J. L. Hoge G. L. Breckenridge J. Sharp-Jeffs N. Chung D. (2020). Economic abuse as an invisible form of domestic violence: A multi-country review. Trauma, Violence, & Abuse, 21(2), 261–283. 10.1177/1524838018764160 29587598

[bibr39-10778012221127727] Pyles L. Banerjee M. M. (2010). Work experiences of women survivors: Insights from the capabilities approach. Affilia, 25(1), 43–55. 10.1177/0886109909354984

[bibr40-10778012221127727] Sanders C. (2015). Economic abuse in the lives of women abused by an intimate partner: A qualitative study. Violence Against Women, 21(1), 3–29. 10.1177/1077801214564167 25548376

[bibr41-10778012221127727] Seith C. (2001). Security matters: Domestic violence and public social services. Violence Against Women, 7(7), 799–820. 10.1177/10778010122182749

[bibr42-10778012221127727] Sharp-Jeffs N. (2015). Money matters: Research into the extent and nature of financial abuse within intimate relationships in the UK. The Cooperative Bank.

[bibr43-10778012221127727] Skinner C. Bradshaw J. Davidson J. (2007). Child support policy: An international perspective. Corporate Document Services.

[bibr44-10778012221127727] Stylianou A. (2018). Economic abuse within intimate partner violence: A review of the literature. Violence and Victims, 33(1), 3-22. 10.1891/0886-6708.33.1.3 29195516

[bibr45-10778012221127727] Stylianou A. M. Postmus J. L. McMahon S. (2013). Measuring abusive behaviors: Is economic abuse a unique form of abuse? Journal of Interpersonal Violence, 28(16), 3186–3204. 10.1177/0886260513496904 23946140

[bibr47-10778012221127727] Surviving Economic Abuse (2021). *Transforming responses to economic abuse*. https://survivingeconomicabuse.org/

[bibr48-10778012221127727] Swanberg J. E. Logan T. K. (2005). Domestic violence and employment: A qualitative study. Journal of Occupational Health Psychology, 10(1), 3–17. 10.1037/1076-8998.10.1.3 15656717

[bibr49-10778012221127727] Tenkorang E. Y. Owusu A. Y. (2019). Does economic abuse affect the health outcomes of women in Ghana? Health Education & Behavior, 46(2), 340–348. 10.1177/1090198118806970 30304953

[bibr50-10778012221127727] Toews M. L. Bermea A. M. (2017). “I was naive in thinking, ‘I divorced this man, he is out of my life’”: A qualitative exploration of post-separation power and control tactics experienced by women. Journal of Interpersonal Violence, 32(14), 2166–2189. 10.1177/0886260515591278 26088900

[bibr51-10778012221127727] Ulmestig R. Eriksson M. (2017). Financial consequences of leaving violent men – women survivors of domestic violence and the social assistance system in Sweden. European Journal of Social Work, 20(4), 560–571. 10.1080/13691457.2016.1188778

[bibr52-10778012221127727] Usta J. Makarem N. N. Habib R. R. (2013). Economic abuse in Lebanon: Experiences and perceptions. Violence Against Women, 19(3), 356–375. 10.1177/1077801213486313 23610198

[bibr53-10778012221127727] Van der Aa S. Niemi J. Sosa L. Ferreira A. Baldry A. (2015). Mapping the legislation and assessing the impact of protection orders in the European Member States. Wolf Legal Publishers.

[bibr54-10778012221127727] Voth Schrag R. J. (2015). Economic abuse and later material hardship: Is depression a mediator? Affilia, 30(3), 341–351. 10.1177/0886109914541118

[bibr55-10778012221127727] Voth Schrag R. J. Edmond T. Tlapek S. M. Auslander W. (2016). Exposure to economically abusive tactics among adolescent girls in the child welfare system. Child and Adolescent Social Work Journal, 34(2), 127–136. 10.1007/s10560-016-0450-8

[bibr56-10778012221127727] Watson L. B. Ancis J. R. (2013). Power and control in the legal system: From marriage/relationship to divorce and custody. Violence Against Women, 19(2), 166–186. 10.1177/1077801213478027 23446105

[bibr57-10778012221127727] Wemrell M. Stjernlöf S. Aenishänslin J. Lila M. Gracia E. Ivert A.-K. (2019). Towards understanding the nordic paradox: A review of qualitative interview studies on intimate partner violence against women (IPVAW) in Sweden. Sociology Compass, 13, e12699. 10.1111/soc4.12699

[bibr58-10778012221127727] Wilcox P. (2006). Surviving domestic violence: Gender, poverty, and agency. Palgrave Macmillan.

[bibr59-10778012221127727] Zeoli A. M. Rivera E. A. Sullivan C. M. Kubiak S. (2013). Post-separation abuse of women and their children: Boundary-setting and family court utilization among victimized mothers. Journal of Family Violence, 28(6), 547–560. 10.1007/s10896-013-9528-7 23956494 PMC3743119

